# Characterization of the complete chloroplast genome of *Wolffia arrhiza* and comparative genomic analysis with relative *Wolffia* species

**DOI:** 10.1038/s41598-024-56394-7

**Published:** 2024-03-11

**Authors:** Halim Park, Jin Hwa Park, Yang Jae Kang

**Affiliations:** 1 Division of Bio and Medical Bigdata Department (BK4 Program), Gyeongsang National University, Jinju, 52828 Republic of Korea; 2 DEEVO Inc., Jinju, 52828 Republic of Korea; 3 Division of Life Science Department at Gyeongsang National University, Jinju, Republic of Korea

**Keywords:** Evolution, Genetics

## Abstract

Lemnoideae, commonly referred to as the duckweed, are aquatic plants found worldwide. *Wolffia* species are known for their extreme reduction in size and complexity, lacking both roots and leaves, and they hold the distinction of being the smallest plants among angiosperms. Interestingly, it belongs to the Araceae family, despite its apparent morphological differences from land plants in the same family. Traditional morphological methods have limitations in classifying these plants, making molecular-level information essential. The chloroplast genome of *Wolffia arrhiza* is revealed that a total length of 169,602 bp and a total GC content of 35.78%. It follows the typical quadripartite structure, which includes a large single copy (LSC, 92,172 bp) region, a small single copy (SSC, 13,686 bp) region, and a pair of inverted repeat (IR, 31,872 bp each) regions. There are 131 genes characterized, comprising 86 Protein-Coding Genes, 37 Transfer RNA (tRNA) genes, and 8 ribosomal RNA (rRNA) genes. Moreover, 48 simple sequence repeats and 32 long repeat sequences were detected. Comparative analysis between *W. arrhiza* and six other Lemnoideae species identified 12 hotspots of high nucleotide diversity. In addition, a phylogenetic analysis was performed using 14 species belonging to the Araceae family and one external species as an outgroup. This analysis unveiled *W*. *arrhiza* and *Wolffia globosa* as closely related sister species. Therefore, this research has revealed the complete chloroplast genome data of *W*. *arrhiza*, offering a more detailed understanding of its evolutionary position and phylogenetic categorization within the Lemnoideae subfamily.

## Introduction

**Figure 1:**
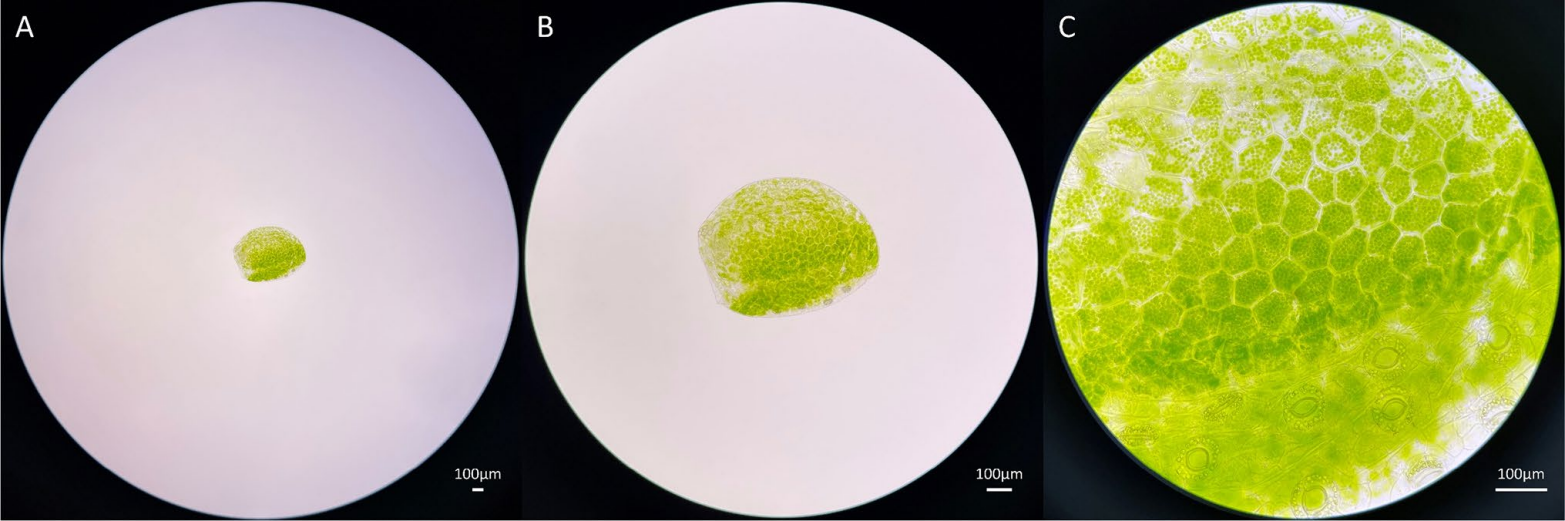
Microscopic photos of *Wolffia arrhiza* taken at (**A**) 40×, (**B**) 100×, and (**C**) 400× magnification. The majority is composed of chloroplast, and stomata can be seen in the lower part of the specimen. The scale bar in the lower right of each figure is 100μm for each magnification.

Lemnoideae, commonly known as duckweed, is a monocotyledonous aquatic plant belonging to the Araceae family^1,2^. It exhibits a growth pattern of floating freely or being submerged^3,4^. It is extensively distributed worldwide, with a particular prevalence in tropical and subtropical regions^1,5–7^. It thrives in freshwater ponds, rivers, and various other aquatic environments. These were classified into a total of five genera and 38 species: *Spirodela* SCHLEID (containing 2 species), *Landoltia* LES & D. J. CRAWFORD (comprising 1 species), *Lemna* L. (with 14 species), *Wolffiella* HEGELM (including 10 species), and *Wolffia* SCHLEID (encompassing 11 species). These findings were initially documented by Landolt in 1986^1,8,9^. Of these, the *Wolffia* genus is notable for being the smallest angiosperm plant in the world, measuring merely 1 mm in diameter, and it does not possess stems or roots. Instead, it has spherical fronds^10^. It features a level upper surface that hovers above the water's top layer, with parallelly arranged stomata (Fig. 1). Additionally, this plant generates infrequently small flowers with only one stamen and pistil, which emerge from a hole on the upper side of the frond. However, the main method of propagation is vegetative reproduction^11,12^. This is the way in which a new leaf bud emerges from the reproductive pouch of the parent leaf, undergoing gradual growth and eventual separation^13^. Consequently, *Wolffia* has the capacity to rapidly double its population in as little as 2–3 days^14,15^ in the optimal conditions such as temperatures within the range of 20–30 °C and a pH level spanning from 5.0 to 7.0^16,17^. This exceptional ability establishes *Wolffia* as one of the fastest-growing plants globally.

Because of its rapid growth, high reproductive rate, and straightforward cultivation and harvesting, it was regarded as an ideal candidate for an experimental model plant in numerous fields. *Wolffia arrhiza*, specifically, has the capacity to absorb and cleanse excessive nitrogen and phosphorus that may result in eutrophication in aquatic environments^18^. Furthermore, its nutritional potential is noteworthy, as 40% of its dry weight is constituted by protein, and it contains notable levels of amino acids, calcium, magnesium, and vitamins that hold significance in the human diet^19^. These characteristics have captured interest, leading to research into the application of *W*. *arrhiza* in fields such as wastewater management^20^, components of human food^21^ or animal feed^22^.

The morphological classification of these duckweed species is exceptionally challenging due to their significantly diminished size and simplified intricacy. Accordingly, molecular taxonomy has been vital for species classification, and to establish the phylogenetic relationships within the Lemnoideae subfamily, the chloroplast barcode has been adopted and utilized^23–25^. In contrast to the nuclear genome, the chloroplast genome presents advantages in species classification owing to its smaller genome size, haploid inheritance, conserved structure, and slower mutation rate^26,27^. This enabled to the classification of Lemnoideae within the Araceae family, alongside land plants that have considerable morphological dissimilarities^28^. Moreover, it has been indicated that *Landoltia*, previously grouped within the *Spirodela* genus, is now a novel and separate genus, distinct from both *Spirodela* and *Lemna*^29^. Thus, in order to establish a robust basis for gaining insight into the genetic variation and its placement in the phylogenetic tree of Lemnoideae subfamily, it is imperative to gather more chloroplast information from a diverse of duckweed species^9,30^.

In this study, the full chloroplast genome of *W*. *arrhiza* was assembled from whole genome sequencing data from MGI DNBSEQ-G50 second-generation sequencing platform. The overview for conducting research is as follows: (i) characterization of the complete chloroplast genome of *W*. *arrhiza*; (ii) a comparative analysis of chloroplast genome data using six species available from NCBI; (iii) Analyzing evolution progression and phylogenetic relationship. The goal is to improve the clarify of relationships within Lemnoideae and establish a fundamental basis for a coherent classification system.

## Results

### Chloroplast genome characteristics of *W*. *arrhiza*

**Figure 2:**
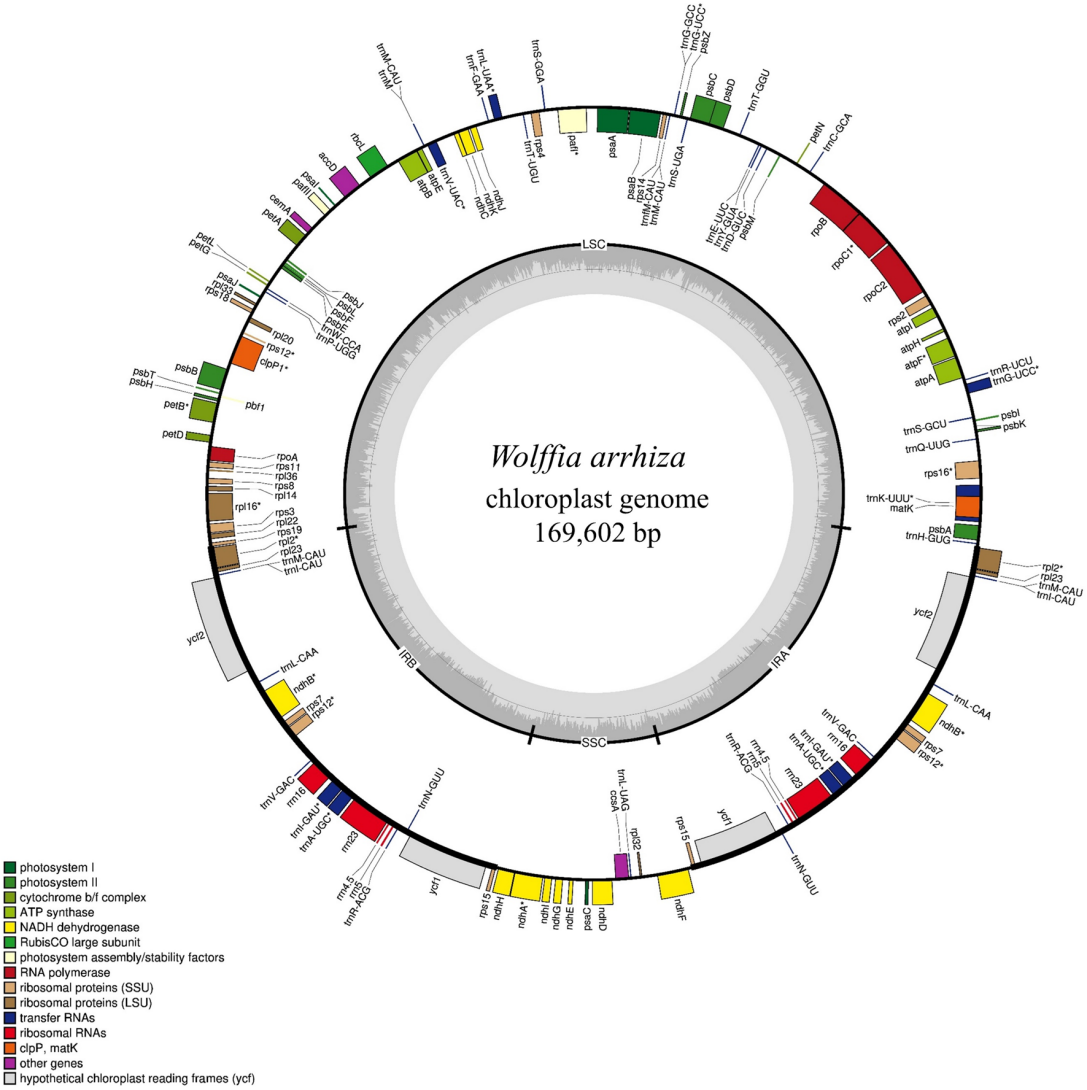
The gene map of the chloroplast genome of *Wolffia arrhiza*. The map identifies three distinct regions: the large single copy region (LSC), the small single copy region (SSC), and the inverted repeat A/B regions (IRA/B). Additionally, the innermost dark gray track represents the GC contents. Genes on the inner side of the map are transcribed counterclockwise, while those on the outer side are transcribed clockwise.

The chloroplast genome of *W. arrhiza* is 169,602 bp in the quadripartite structure with one large single copy (LSC) region of 92,172 bp, one small single copy (SSC) region of 13,686 bp, and a pair of inverted repeat (IR) regions of 31,872 bp each (Fig. 2). The total guanine and cytosine (GC) content of the chloroplast genome is 35.78%. The relative occupation ratio of the LSC, SSC, and IR regions in the chloroplast genome were 33.63%, 30.79%, and 39.97%, respectively. It contains a total of 131 predicted genes, which are divided into three groups: 86 protein-coding genes (PCGs), 37 transfer RNA (tRNA) genes, and 8 ribosomal RNA (rRNA) genes. The entire set of genes exhibited a GC content of 38.52%. Within this, PCGs demonstrated a GC content of 37.08%, tRNA genes had a GC content of 52.50%, and rRNA genes exhibited a GC content of 54.69%. The LSC region contained a total of 83 genes, comprising 61 PCGs and 22 tRNA genes. In the SSC region, there were 11 genes, including 10 PCGs and one tRNA gene. The IR regions consisted of 36 genes, with seven PCGs, seven tRNA genes, and four rRNA genes duplicated (Table 1). Additionally, the *rps12* gene is a trans-spliced gene that exons found in both the LSC and IRs, while the *rps19* gene extended across two regions between the LSC and IRb.

**Table 1:** Chloroplast genome structure and feature of *Wolffia arrhiza.*

Genome feature		Length (bp)/Numbers	GC content (%)
Structure length	Total	169,602	35.78
	LSC region	92,172	33.63
	SSC region	13,686	30.79
	IR (a/b) region	31,872	39.97
Counts of genes in different categories	Genes	131	38.52
	PCGs	86	37.08
	tRNA	37	52.50
	rRNA	8	54.69
Detailed counts of genes in different regions	LSC region	61 PCGs, 22 tRNA	–
	SSC region	10 PCGs, 1 tRNA	–
	IR (a/b) region	7 PCGs, 7 tRNA, 4 rRNA	–

According to the chloroplast genome annotation of *W*. *arrhiza*, 112 unique genes were categorized into four functional groups. There were 59 transcription and translation-related genes, 46 photosynthesis-related genes, five biosynthesis-related genes, and two genes whose functions were unidentified (Table 2).

**Table 2:** Genetic classification of the chloroplast genome of *Wolffia arrhiza*.

Category (number)	Group (number)	Gene name
Transcription and translation (59)	Ribosomal RNAs (4)	rrn16*, rrn23*, rrn4.5*, rrn5*
	Transfer RNAs (30)	*trnA*-UGC*, *trnC*-*GCA*, *trnD*-*GUC*, *trnE*-*UUC*, *trnF*-*GAA*, *trnG*-*GCC*, *trnG*-*UCC*, *trnH*-*GUG*, *trnI*-CAU*, *trnI*-GAU*, *trnK*-*UUU*, *trnL*-CAA*, *trnL*-*UAA*, *trnL*-*UAG*, *trnM*-*CAU*, *trnN*-GUU*, *trnP*-*UGG*, *trnQ*-*UUG*, *trnR*-ACG*, *trnR*-*UCU*, *trnS*-*GCU*, *trnS*-*GGA*, *trnS*-*UGA*, *trnT*-*GGU*, *trnT*-*UGU*, *trnV*-GAC*, *trnV*-*UAC*, *trnW*-*CCA*, *trnY*-*GUA*, *trnfM*-*CAU*
	Small subunit of ribosome (SSU) (12)	*rps2*, *rps3*, *rps4*, rps7*, *rps8*, *rps11*, rps12*, *rps14*, rps15*, *rps16*, *rps18*, *rps19*
	Large subunit of ribosome (LSU) (9)	rpl2*, *rpl14*, *rpl16*, *rpl20*, *rpl22*, rpl23*, *rpl32*, *rpl33*, *rpl36*
	DNA-dependent RNA polymerase (4)	*rpoA*, *rpoB*, *rpoC1*, *rpoC2*
Photosynthesis (46)	Photosystem I (7)	*psaA*, *psaB*, *psaC*, *psaI*, *psaJ*, *pafI*, *pafII*
	Photosystem II (15)	*psbA*, *psbB*, *psbC*, *psbD*, *psbE*, *psbF*, *psbH*, *psbI*, *psbJ*, *psbK*, *psbL*, *psbM*, *psbT*, *psbZ*, *pbf1*
	Subunit of cytochrome (6)	*petA*, *petB*, *petD*, *petG*, *petL*, *petN*
	ATP synthase (6)	*atpA*, *atpB*, *atpE*, *atpF*, *atpH*, *atpI*
	RubisCO (1)	*rbcL*
	NADH dehydrogenase (11)	*ndhA*, ndhB*, *ndhC*, *ndhD*, *ndhE*, *ndhF*, *ndhG*, *ndhH*, *ndhI*, *ndhJ*, *ndhK*
Biosynthesis (5)	Maturase (1)	*matK*
	ATP-dependent Protease (1)	*clpP1*
	Acetyl-CoA-carboxylase (1)	*accD*
	Envelop membrane protein (1)	*cemA*
	C-Type cytochrome synthesis (1)	*ccsA*
Unknown (2)	Hypothetical chloroplast reading frames(ycf) (2)	ycf1*, ycf2*

Duplicated genes are denoted by an asterisk (*).

Concurrently, a total of 17 unique intron genes were detected, and they were distributed across the LSC (11), IR (5), and SSC (1, *ndhA*) regions. It was comprised 11 PCGs and 6 tRNA genes. Among these, 15 genes (*atpF*, *ndhA*, *ndhB*, *petB*, *rpl16*, *rpl2*, *rpoC1*, *rps12*, *rps16*, *trnA*-*UGC*, *trnG*-*UCC*, *trnI*-*GAU*, *trnK*-*UUU*, *trnL*-*UAA*, *trnV*-*UAC*) had a single intron each, while the remaining 2 genes (*clpP1*, *pafI*) contained two introns each (Table 3).

**Table 3:** Introns and exons length information of the *Wolffia arrhiza*.

Gene	Location	Exon I (bp)	Intron I (bp)	Exon II (bp)	Intron II (bp)	Exon III (bp)
*atpF*	LSC	144	839	402		
clpP1*	LSC	71	811	295	656	243
*ndhA*	SSC	551	916	532		
ndhB*	IR	777	703	756		
pafI*	LSC	126	750	226	789	155
*petB*	LSC	6	752	642		
*rpl16*	LSC	9	1575	399		
rpl2*	IR	391	660	431		
*rpoC1*	LSC	453	744	1620		
rps12*	LSC, IR	114	540	232	–	26
*rps16*	LSC	40	1025	200		
*trnA*-UGC*	IR	37	801	36		
*trnG*-*UCC*	LSC	24	619	48		
*trnI*-GAU*	IR	42	804	35		
*trnK*-*UUU*	LSC	36	2545	43		
*trnL*-*UAA*	LSC	37	507	50		
*trnV*-*UAC*	LSC	38	615	37		

Duplicated genes are denoted by an asterisk (*). The duplicated 3' ends of the *rps12* gene, which is trans-spliced, are found in the IR regions with the 5' end of the gene located in the LSC region.

### Repeat sequences analysis

The web application Misa successfully identified a total of 48 Simple Sequence Repeats, SSRs, with lengths ranging from 10 to 16 bp. There was a total of 42 mononucleotide and 6 dinucleotide repeat types observed, all composed of A or T bases. There were 26 mononucleotides consisting solely of A and 16 mononucleotides consisting solely of T. Likewise, four dinucleotides comprised of AT repeats and two dinucleotides comprised of TA repeats. Among the identified SSRs, the LSC region contains the highest number, accounting for the majority (72.92%) with a total of 35 SSRs. The SSC region hosts seven SSRs (14.58%), while the IR region holds six SSRs (12.5%). At the same time, the Intergenic spacer (IGS) region presents the largest number of SSRs, totaling 40 (83.3%) of the total SSRs. Five SSRs (10.42%) were found in introns, and the remaining three SSRs (6.25%) were in PCG regions. Notably, each of the introns within the *petB*, *rps16*, *trnK*-*UUU*, *pafI*, and *clpP1* genes contained one SSR. Moreover, SSRs within PCG were observed in one instance within the *rpoB* gene and one each in the *ycf1* genes of IRa and IRb (Table 4).

**Table 4:** The types of SSRs in *Wolffia arrhiza* and their corresponding regions and locations.

Repeat type	Repeat unit	Number of repeats	Repeat length	Number of SSRs	Region	Location
Mono-nucleotide	A	10	10	18	LSC(15)	IGS(12)
						Intron(3, *petB*, *rps16*, *trnK*-*UUU*)
					SSC(3)	IGS(3)
		11	11	5	LSC(2)	IGS(2)
					SSC(1)	IGS(1)
					IRb(2)	IGS(1)
						PCG(1, *ycf1*)
		13	13	3	LSC(2)	IGS(2)
					IRb(1)	IGS(1)
	T	10	10	9	LSC(7)	IGS(5)
						Intron(1, *pafI*)
						PCG(1, *rpoB*)
					SSC(2)	IGS(2)
		11	11	5	LSC(3)	IGS(2)
						Intron(1, *clpP1*)
					IRa(2)	IGS(1)
						PCG(1, *ycf1*)
		12	12	1	LSC(1)	IGS(1)
		13	13	1	IRa(1)	IGS(1)
Di-nucleotide	AT	6	12	3	LSC(3)	IGS(3)
		7	14	1	LSC(1)	IGS(1)
	TA	6	12	1	SSC(1)	IGS(1)
		8	16	1	LSC(1)	IGS(1)
Total				48	LSC(35, 72.92%) SSC(7, 14.58%) IR(6, 12.5%)	IGS(40, 83.3%) Intron(5, 10.42%) PCG(3, 6.25%)

A total of 32 long repeat sequences were detected using the REPuter web application. Among these repeats, there were 16 forward repeats (F), 1 reverse repeat (R), and 15 palindromic repeats (P). The lengths exhibited a distribution ranging from 30 to 69 bp, and among them, a unique palindromic repeat measuring 31,872 bp in length was identified. Out of these, 13 were exclusively located within the LSC region (40.63%), while 6 were uniquely situated in the IR region (18.75%). Additionally, 7 were suspended across both IRa and IRb (21.87%), with the remaining 6 spanning across the LSC and IR (18.75%), covering two structural regions. Furthermore, there were a total of 12 repeats solely present within a single PCG (37.5%), and all these repeats were located in the *ycf2* gene. There was also one repeat that distributed across both the intron and the PCG (3.12%), and it was the longest repeat in terms of length. This was present across a total of 20 genes. One repeat was identified in both the IGS and the PCG (3.12%), and PCG corresponded to the *pbf1* gene. There were three repeats spanning two PCGs (9.38%), and all these PCGs were identified as tRNA genes. In addition, six repeats were observed, spanning across introns and the IGS (18.75%), with four of them positioned in introns within the *pafI* gene, and the other two in the *petB* gene. The remaining nine repeats, containing the only reverse repeat that was detected, were exclusively located in the IGS (28.13%) (Table 5).

**Table 5:** The types of long repeat in *Wolffia arrhiza* and their corresponding regions and locations.

Repeats match type	Repeats length range	Repeats length	Number of repeats set	Region of repeats set	Location of repeats set
F	30–39	30	4	LSC(3)	IGS(2)
					PCG(1, *trnS*(*GCU*)-*trnS*(*UGA*))
				LSC-IRb(1)	Intron-IGS(1, *pafI*)
		31	3	LSC(1)	IGS(1)
				IRa(1)	PCG(1, *ycf2*)
				IRb(1)	PCG(1, *ycf2*)
		32	1	LSC(1)	IGS(1)
		34	1	LSC(1)	IGS(1)
		36	1	LSC-IRa(1)	Intron-IGS(1, *petB*)
		37	2	IRa(1)	PCG(1, *ycf2*)
				IRb(1)	PCG(1, *ycf2*)
		39	1	LSC-IRb(1)	Intron-IGS(1, *pafI*)
	40–49	41	2	IRa(1)	PCG(1, *ycf2*)
				IRb(1)	PCG(1, *ycf2*)
	60–69	63	1	LSC(1)	IGS(1)
P	30–39	30	2	LSC(1)	IGS(1)
				LSC-IRa(1)	Intron-IGS(1, *pafI*)
		31	4	LSC(2)	IGS(1)
					IGS-PCG(1, *pbf1*)
				IRa-IRb(2)	PCG(2, *ycf2*)
		32	2	LSC(2)	PCG(2, *trnS*(*GCU*)-*trnS*(*GGA*) / *trnS*(*UGA*)-*trnS*(*GGA*))
		36	1	LSC-IRb(1)	Intron-IGS(1, *petB*)
		37	2	IRa-IRb(2)	PCG(2, *ycf2*)
		39	1	LSC-IRa(1)	Intron- IGS(1, *pafI*)
	40–49	41	2	IRa-IRb(2)	PCG(2, *ycf2*)
	70~	31,872	1	IRa-IRb(1)	Intron-PCG(1, *rps7*-*ycf2*-*ndhB*-*ycf1-trnA*(*UGC*)-*rpl2-rpl23*-*trnI*(*CAU*)-*rrn5*-*rrn23*-*rrn16*-*trnN*(*GUU*)-*trnV*(*GAC*)-*trnR*(*ACG*)-*rps12*-*rrn4.5*-*rps15*-*trnI*(*GAU*)-*trnL*(*CAA*)- *trnM*(*CAU*)
R	30–39	32	1	LSC(1)	IGS(1)

In the case of differing regions or locations between the repeats, they are connected using '-' and indicated as a repeat set. The genes follow the same pattern. F for forward repeat, R for reverse repeat, and P for palindromic repeat.

### Codon usage

A total of 86 PCGs and their CDS were extracted from the chloroplast genome of *W*. *arrhiza*. These sequences have a combined length of 84,507 bp and consist of 28,169 codons. Leucine (Leu) was the most commonly encoded amino acid, comprising 10.50% of the total with 2959 codons. Conversely, Cysteine (Cys) was the least frequently encoded amino acid, making up only 1.10% of the total with 310 codons. The RSCU values for each codon fell within the range of 0.3 (CGG, Arg) to 2.01 (AGA, Arg). Out of a total of 30 codons with a high frequency of usage (RSCU > 1), except for UUG (Leu), 29 of these preferred synonymous codons ended with A or U(T) nucleotides. For the 32 codons with RSCU < 1, the 29 codons ended with C or G nucleotide, excluding CUA(Leu), AUA(Ile), and UGA(TER). Additionally, the terminator most preferred was UAA, showing an RSCU value of 1.60. In contrast, the codons AUG (Met) and UGG (Trp) demonstrated an RSCU value of 1, suggesting there is no bias as they each encode only one amino acid (Table S1).

### Comparison of chloroplast genomes within Lemnoideae

**Figure 3:**
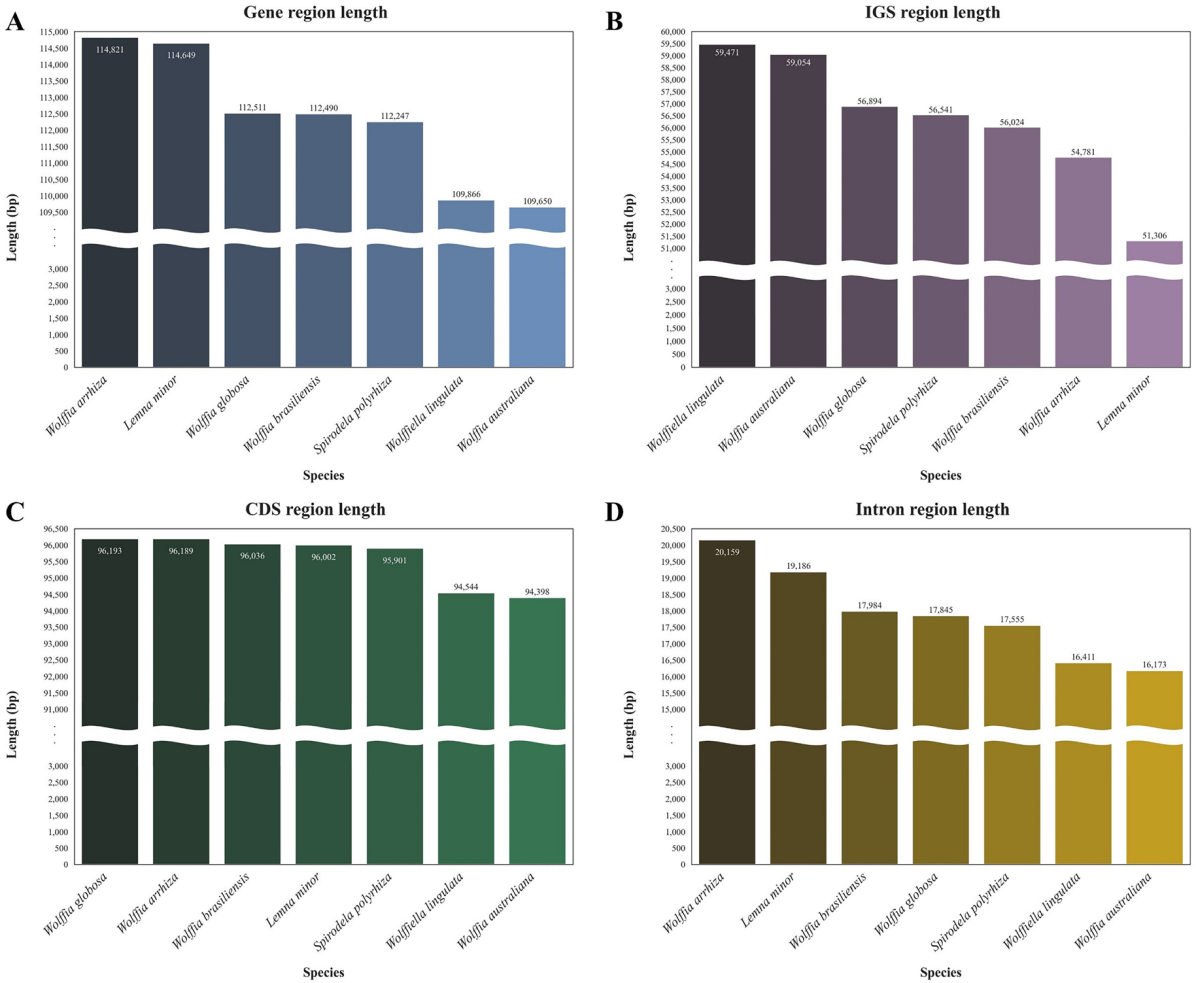
Length for each region of the Lemnoideae, including *Wolffia arrhiza*. (**A**) Gene region length (**B**) IGS region length (**C**) CDS region length (**D**) Intron region length. The X-axis represents the species names, while the Y-axis depicts the lengths of the regions. The arrangement of each graph is based on the ascending order of region lengths.

The lengths of genes and IGS regions were compared among the chloroplast genomes of seven species of duckweed within the Lemnoideae subfamily (Fig. 3). The gene regions exhibited a range in length from 109,650 bp to 114,821 bp. Among the seven Lemnoideae species, *W. arrhiza* possessed the longest gene region with 114,821 bp (Fig. 3A). On the other hand, IGS regions had lengths that ranged from 51,306 bp to 59,471 bp, with *W. arrhiza* possessing the second shortest IGS region at 54,781 bp, following *Lemna minor* (Fig. 3B). The gene regions were further categorized into coding sequences (CDS) and intron regions. It was observed that CDS regions had a length range from 94,398 bp to 96,193 bp. Notably, *W. arrhiza* possessed the second-longest CDS region, measuring 96,189 bp, which was 4 bp shorter than *W. globosa* (Fig. 3C). Conversely, the lengths of intron regions ranged from 16,173 bp to 20,159 bp, with *W. arrhiza* having the longest intron region at 20,159 bp (Fig. 3D).

**Figure 4:**
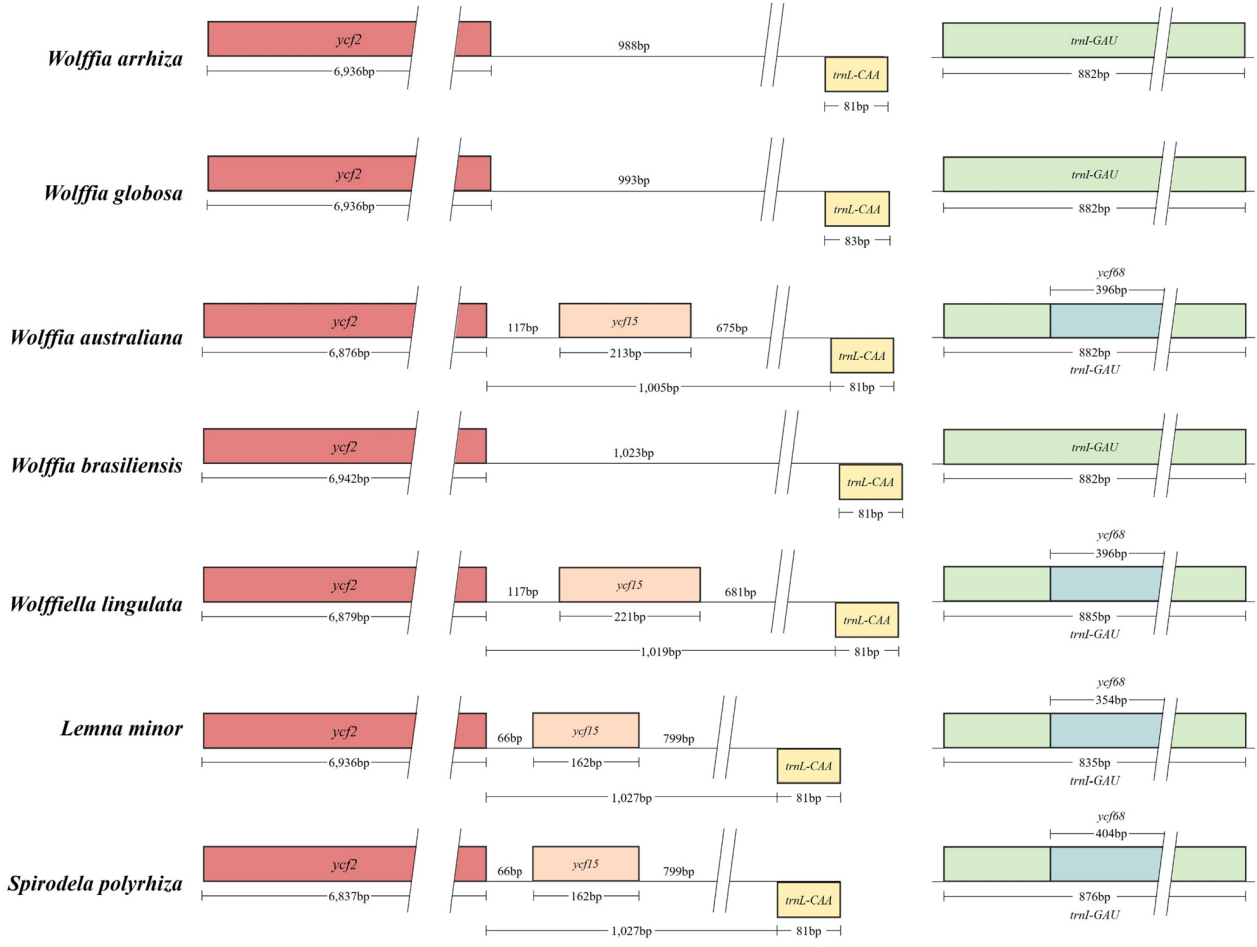
Comparative analysis of alterations resulting from the *ycf68*, and *ycf15* deletion in *Wolffia arrhiza*. This represents one of the IR regions, and the other IR region exhibits a same aspect with reverse complementarity. The squares represent genes, where those transcribed on the forward strand are positioned at the top of the line, and those transcribed on the reverse strand are located at the bottom of the line. The numbers adjacent to the squares represent the lengths of individual genes, while the numbers above the lines are the lengths of IGS between each gene. *ycf2* is depicted by the color red, *ycf15* by orange, *trnL*-*CAA* by yellow, *trnI*-*GAU* by green, and *ycf68* by blue.

To gain further insight into these changes, an analysis was conducted on events such as insertions, deletions, duplications, and intron changes in genes across Lemnoideae species (Table S2). Compared to other Lemnoideae species, *W. arrhiza* was represented by the genes *pafI*, *pafII*, *clpP1*, and *pbfI*, which are synonymous with the *ycf3*, *ycf4*, *clpP*, and *psbN* genes in other species. When comparing other genes, the most significant alterations were the deletion events of pseudogenes *ycf68* and *ycf15* in the IR region (Fig. 4). Upon a more detailed examination, it was observed that the gene *ycf68*, which had perfect overlap with *trnI*-*GAU* in other species, was deleted in *W. arrhiza*, leaving only *trnI*-*GAU*. However, another deleted gene, *ycf15*, which has been alone in other species, underwent deletion in *W. arrhiza* and sequences remained at an IGS region. The length between *ycf2* and *trnL*-*CAA* flanking *ycf15* in *W. australiana*, *Wolffiella lingulata*, *Lemna minor*, and *Spirodela polyrhiza* were 1005 bp, 1019 bp, 1027 bp, and 1027 bp, respectively. In *W. arrhiza*, *W. globosa*, and *W. brasiliensis*, where *ycf15* was deleted, the IGS length between *ycf2* and *trnL*-*CAA* was 988 bp, 993 bp, and 1023 bp, respectively.

To identify variations in introns, the gap between the longest and shortest length values for each species within the same gene was calculated (Table S2). As a result, it was determined that the lengths of the *petB* and *rpl16* genes in *W*. *arrhiza* are 1400 bp and 1983 bp, respectively. These lengths exceed twice the sizes observed in other species, where they typically range from 642 to 701 bp for *petB* and 411 bp (the exception of *Lemna minor*, which has a length of 1714 bp) for *rpl16*. When analyzing the exons and introns of these genes in each species, the exons exhibit consistent lengths across all species (ranging from 642 to 654 bp for *petB* and 408–411 bp for *rpl16*). However, in *W*. *arrhiza*, the introns are notably longer, with the *petB* gene containing a 752 bp intron and the *rpl16* gene containing a 1575 bp intron (Table S3).

**Figure 5:**
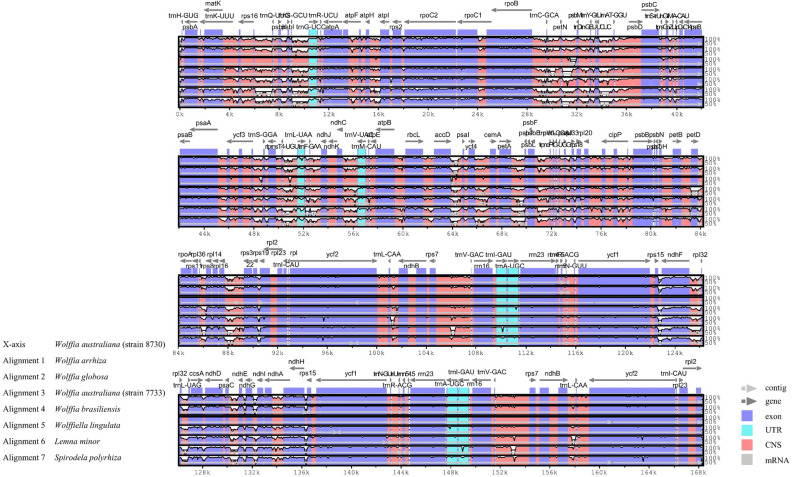
Analyzed the chloroplast genome sequences of seven Lemnoideae species, including *Wolffia arrhiza*, using mVista, with *Wolffia australiana* as the reference. The X-axis represents the coordinates of the chloroplast genome sequence position, while the Y-axis indicates the range of sequence identity from 50 to 100%. The direction and position of the genes are depicted by the gray arrows on the graph. The graph's shaded colors have the following meanings: the dark blue regions correspond to protein coding sequences (CDS), the pink regions represent Conserved Non-Coding Sequences (CNS), and the light-blue regions indicate UTRs.

Sequence homology among the chloroplast genomes of seven species within the Lemnoideae subfamily was assessed and visualized via the shuffle-LAGAN mode in mVista. The annotation data relied upon the reference strain, *W*. *australiana* (strain 8730). As a result, it was established that the chloroplast genome sequence of duckweed maintains a high degree of sequence conservation, with very few regions exhibiting sequence identity below 90% (Fig. 5). In detail, the IR region showed a higher level of preservation when contrasted with the LSC and SSC regions. In addition, the mutation rate was greater in the IGS region in contrast to the PCG region. The majority of PCGs were generally well-preserved, but significant variations were observed in some PCGs, including *matK*, *rpoC2*, *ndhF*, *cssA*, *ndhD*, and *ndhH*. In contrast to the PCG regions, the non-coding regions showed a relatively higher mutation rate in numerous locations. Within non-coding regions, intergenic regions displayed the highest variability rate. Upon visual examination of the figure, the most notable segments appeared to be *trnC*(*GCA*)-*petN*, *petN*-*psbM*, and *trnE*(*UUC*)-*trnT*(*GGU*).

**Figure 6:**
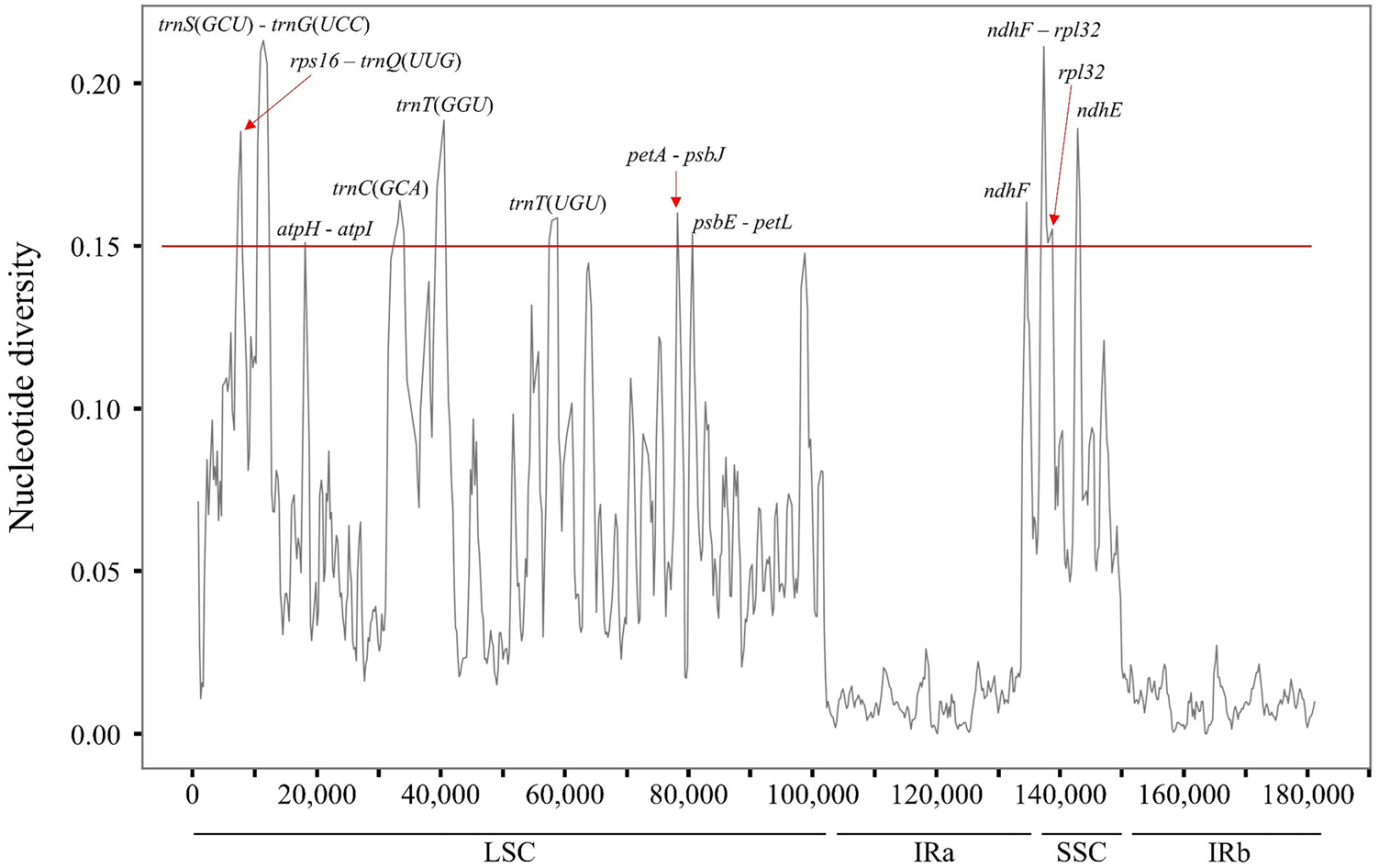
Nucleotide diversity of chloroplast genome sequences in Lemnoideae, including *Wolffia arrhiza*. The X-axis represents the alignment sequence's position, while the Y-axis indicates the values for nucleotide diversity. The use of a hyphen to connect two genes signifies a non-coding region, whereas the representation of a single gene indicates a coding region.

To clearly identify the variable regions within the mVista results, a sliding window analysis was executed using DnaSP v.6.10 software, followed by the calculation of nucleotide diversity values (π, Pi). There were 769 nucleotide diversity point observed, with values ranging from 0.00000 to 0.21294, and an average value of 0.04589 (Fig. 6). The nucleotide diversity value was highest (0.21294) in the LSC region, while the IR region had the lowest value (0.00048), excluding zero. In this regard, the IR region exhibited significantly lower variability compared to the LSC and SSC regions. Among them, 12 locations demonstrated high Pi values greater than 0.15. Eight of them were found in the LSC region, while four were located in the SSC region. Within the LSC region, 5 locations were detected in intergenic regions including *rps16*-*trnQ*(*UUG*), *trnS*(*GCU*)-*trnG*(*UCC*), *atpH*-*atpI*, *petA*-*psbJ*, *psbE*-*petL*, while 3 locations were found in the coding regions of *trnC*(*GCA*), *trnT*(*GGU*), and *trnT*(*UGU*). In the SSC region, one of the four positions was located in the intergenic region of *ndhF*-*rpl32*, whereas the other three were found in the coding regions of *ndhF*, *rpl32*, and *ndhE*. The coding region and non-coding region with the highest nucleotide diversity values were *trnT*(*GGU*) (0.18841) and *trnS*(*GCU*)-*trnG*(*UCC*) (0.21294), respectively, located in the LSC.

To delve deeper into the nucleotide diversity results, SNPs and InDels were analyzed in seven Lemnoideae species, utilizing *Wolffia australiana* as a reference. This revealed 17,269 SNPs and 2030 InDels. The majority of SNPs appeared in the IGS regions (51.91%), followed by exon regions (35.20%) and intron regions (12.89%) (Table S6). These findings aligned with earlier results (Fig. 6), particularly noting that the IGS in *trnS*(*GCU*)—*trnG*(*UCC*) represented 5.30% of the total IGS region, and the IGS in *ndhF*—*rpl32* comprised 4.03% of the total IGS region. Additionally, InDels were predominantly distributed in IGS regions (76.35%), with lesser occurrences in intron (17.10%) and exon regions (6.55%) (Table S7). Most were short InDels of 10 base pairs or fewer, accounting for 80.59% of the total, and one long InDel of 1000 bp was detected. Similarly with SNPs aspect, the IGS regions in *trnS*(*GCU*)—*trnG*(*UCC*), and *ndhF*—*rpl32*, constituted 4.54% and 1.86% of the total IGS region, respectively.

**Figure 7:**
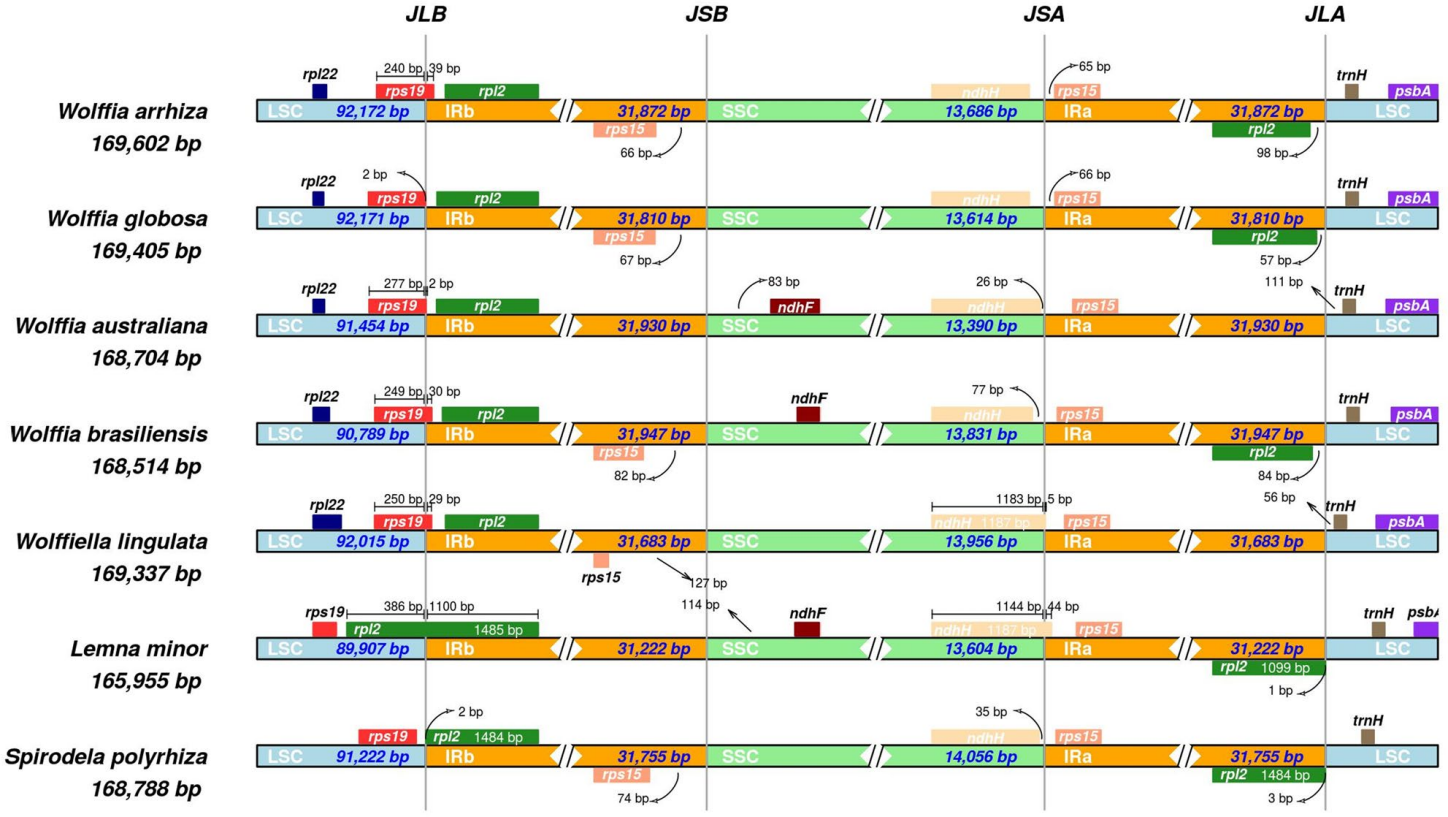
Comparing the boundaries of chloroplast genome regions in seven species, including *Wolffia arrhiza*, from the Lemnoideae subfamily: LSC, IRs, and SSC. The junctions between each pair of genomic regions are indicated as JLB (LSC/IRB), JSB (SSC/IRB), JSA (SSC/IRA), and JLA (LSC/IRA). Genes transcribed on the forward strand are depicted above the line, whereas genes transcribed on the reverse strand are exhibited below the line. Furthermore, the numbers above the genes signify the gap between the gene's start or end and the region's boundary.

The gene distribution at the boundaries of the LSC/SSC and IR regions in the chloroplast genomes of the seven species was compared using IRscope. Overall, the distribution of genes at each boundary region appeared to be similar, with *rpl22*, *rps19*, *rpl2*, *rps15*, *ndhF*, *ndhH*, *trnH*, and *psbA*. However, it was observed that the *rpl2* gene is found solely in the IRb region and is absent in the IRa region of *W*. *australiana* and *Wolffiella lingulata* (Fig. 7, Table S5). Although not shown in the figure due to their location at the boundaries and greater distance, other genes did not undergo any loss. Nevertheless, variations were noted in the association between genes and the boundary lines. The JLB (LSC/IRB) boundary displayed three different configurations: positioned within the *rps19* gene, within the *rpl2* gene, or within IGS between the *rps19* and *rpl2* genes. For *W*. *arrhiza*, the JLB boundary can be found within the *rps19* gene. The *rps19* gene spans 240 bp in the LSC region, and the remaining 39 bp extend into the IRB region. Similar cases are apparent in the *W*. *australiana*, *W*. *brasiliensis*, and *Wolffiella lingulata*. These species have respectively occupied 277 bp, 249 bp, and 250 bp within the LSC region, along with extensions of 2 bp, 30 bp, and 29 bp into the IRB. In the case of *Lemna minor*, it was observed that the boundary of the JLB was located within the *rpl2* gene. The *rpl2* gene spanned 1100 bp within the IRB region, with the remaining 386 bp extending towards the LSC region. *W*. *globosa* and *Spirodela polyrhiza* were both found to have the JLB boundary positioned within the IGS between the *rps19* and *rpl2* genes. Additionally, the *rps19* and *rpl2* genes were identified within the LSC and IRB regions, respectively. In the instance of the JSA (SSC/IRA) boundary, it presented in two different cases, with one found within the *ndhH* gene and the other within the IGS region lying between the *ndhH* and *rps15* genes. For *W*. *arrhiza*, the *ndhH* and *rps15* genes were contained within their respective SSC and IRA regions, rather than extending beyond them. This same pattern was also observed in *W*. *globosa*, *W*. *australiana*, *W*. *brasiliensis*, and *Spirodela polyrhiza*. Nevertheless, for *Wolffiella lingulata* and *Lemna minor*, the boundary of the JSA was positioned within the *ndhH* gene, with each extension 1183 bp and 1144 bp into the SSC region, while the remaining 5 bp and 44 bp entered the IRA region.

### Phylogenetic analysis

**Figure 8:**
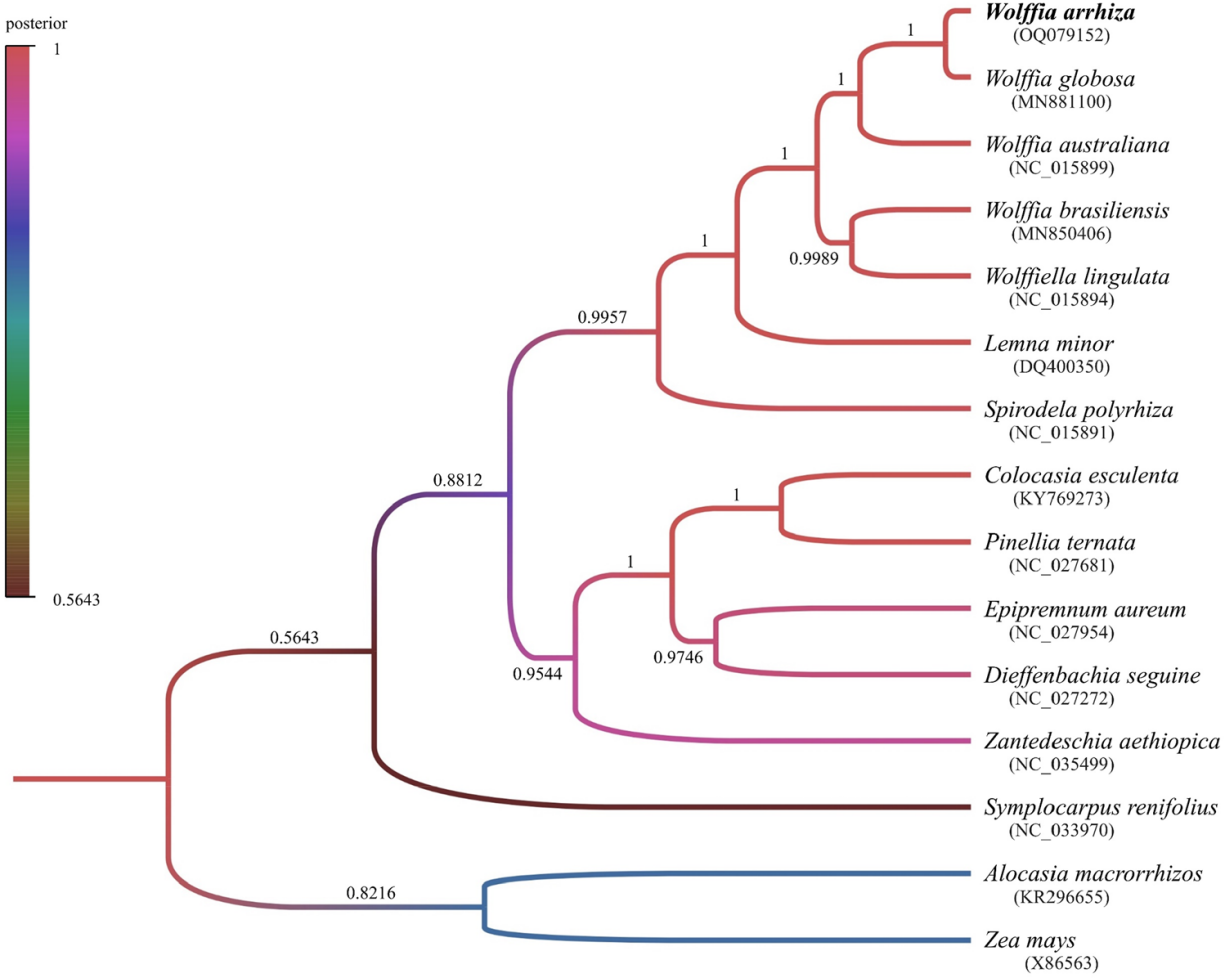
Bayesian phylogenetic tree of Araceae species based on the chloroplast genome data. The colors of the branches in the tree represent the Bayesian posterior probability, as indicated by the color bar. The numerical values displayed on the branches represent precise posterior values. At the bottom of each species name is the genbank accession number.

To explore the phylogenetic relationship of *W*. *arrhiza*, a phylogenetic tree was created that included a total of 15 species. This species set comprised 7 species from the Lemnoideae subfamily, which includes *W*. *arrhiza*, and 7 species within the Araceae family to which Lemnoideae subfamily belongs, along with one outgroup species, *Zea mays*. Excluding genes in the IR, there are 44 PCGs shared between them (Table S4). In addition, based on the results from Prottest, the CpREV + G + I model was determined to be the best fit model for explaining protein evolution across the 15 species. The Bayesian analysis was performed using BEAST v1.10.4 software, employing 50,000,000 Markov Chain Monte Carlo (MCMC) chains with the previously identified shared PCG and the most suitable model. Consequently, the phylogenetic tree was constructed, with Bayesian posterior probability values that ranged from 0.5643 to 1 (Fig. 8). The tree can be divided into three parts: *W*. *arrhiza*—*Spirodela polyrhiza*, *Colocasia esculenta*—*Symplocarpus renifolius*, and the outgroup. *W*. *arrhiza* is classified as part of the *W*. *arrhiza*—*Spirodela polyrhiza* section, belonging to the Lemnoideae subfamily. It exhibits the nearest evolutionary relationship with *W*. *globosa*.

## Discussion

The complete chloroplast genome of *W*. *arrhiza* was assembled using the short reads from MGI DNBSEQ-G50 next-generation sequencing platform, revealing a common circular quadripartite genomic structure. Despite *W*. *arrhiza* is an aquatic plant, it exhibited a similar pattern to land plants, having a length of 169,602 bp falling within the range of 130–170 kbp and a GC content of around 35.78%, which is in proximity to the average of 36.3% in land plants^31^. Additionally, the analysis of SSRs indicated that in *W*. *arrhiza*, SSRs are composed exclusively of A or T nucleotides, predominantly distributed in the LSC region out of the four regions (LSC, SSC, IRs) and in the IGS among the three locations (IGS, PCG, intron) (Table 4). A strong A/T bias and high concentration within the LSC region and IGS were similar to patterns observed in other angiosperms chloroplast genome^32–34^. The analysis of codon usage revealed that all but one of the codons with RSCU values exceeding 1 ended in either A or T (Table S1). This corresponds with research results indicating a preference for A/T-ending codons among synonymous codons, as it offers advantages in codon optimization during gene expression compared to codons ending in C/G^35^. Codon usage patterns are fundamentally dependent on the gene sequences, thereby mirroring nucleotide substitutions that lead to variations of gene, protein structure, and function. Consequently, by understanding codon usage, it was possible to gain a foundational framework for insights into the history of *W*. *arrhiza* in the context of genome evolution^36^.

The analysis of genome information for the Lemnoideae subfamily, including *W*. *arrhiza*, was conducted to identify the various changes that occurred during evolution. First, diversity within the Lemnoideae subfamily, including *W*. *arrhiza*, was analyzed at the nucleotide level, and a total of 12 hotspots were detected. There were several peaks in the data with significantly high Pi values, such as *trnS*(*GCU*)-*trnG*(*GCC*) (0.21294)^37^, *rps16*-*trnQ*(*UUG*) (0.18500)^38^, and *ndhF*-*rpl32* (0.19456)^39^, indicating a substantial nucleotide variability in other angiosperm plants. One can surmise that these genes are inclined to undergo rapid nucleotide substitutions, suggesting at their applicability as molecular markers for phylogenetic analysis and species identification. Subsequently, within the Lemnoideae, it was confirmed that *W*. *arrhiza* had the longest chloroplast genome length among its fellow species (Table S5). Various elements influencing chloroplast length have been reported as the contraction and expansion of the IR region, gene insertions, deletions, duplications, inversions, and alterations in introns^40–43^. In the case of *W*. *arrhiza*, the JLB and JSA boundaries were positioned similarly to most other Lemnoideae species. There was no noticeable expansion or contraction in the IR region, which was 31,872 bp in size. This falls within the average IR range of 31,223 to 31,930 bp observed in other species (Fig. 7 and Table S5). To explore whether there were additional alterations, such as gene rearrangements or inversions, a synteny analysis of the Lemnoideae chloroplast genome was performed utilizing the MAUVE program^44^. The seven Lemnoideae species visually demonstrated a highly similar gene position pattern, suggesting a significant level of collinearity (Fig. S1). The events such as the deletion of *ycf68* and *ycf15*, resulted in changes to the types and number of genes (Fig. 4). However, they could not be considered to have had a significant impact on the length of the whole chloroplast genome. On the other hand, when comparing the lengths of genes, CDS, introns, and intergenic regions, there was a noticeable increase in the total intron length (Fig. 3). This suggests that intron evolution may have occurred in the *W*. *arrhiza* chloroplast genome. As a result, significant alterations were identified in the intron analysis of *petB* and *rpl16* showing from 642–701 bp to 1400 bp, and from 411 to 1983 bp, respectively (Tables S2, S3). While the exact reasons for the insertion of these introns require additional exploration, it implied the significance of both the *petB* and *rpl16* genes. From this, it can be inferred that changes in introns impacted the chloroplast genome length of *W*. *arrhiza*, resulting in it possessing the longest genome length among Lemnoideae species. Additionally, the alterations in introns were commonly detected in various angiosperms, indicating their potential utility as molecular markers for conducting phylogenetic studies and identifying different species^45–49^.

The chloroplast, which contains all of this information, was used to explore the phylogenetic history of the species. The phylogenetic tree was partitioned into three sections: *W*. *arrhiza*—*Spirodela polyrhiza*, *Colocasia esculenta*—*Symplocarpus renifolius*, and the outgroup (Fig. 8, Table S4). In the *W*. *arrhiza*—*Spirodela polyrhiza* section, the majority of posterior values were 1.0, providing a strong representation of the Lemnoideae subfamily lineage. Specifically, the relationship between *W*. *arrhiza* and *W*. *globosa* is affirmed with a posterior value of 1, signifying that they are sister species. *W*. *brasiliensis* showed unclear taxonomic results that did not align with the same genus, *Wolffia*, as observed in previous research^8,9,23^. Nevertheless, through conducting a phylogenetic classification of the Lemnoideae subfamily, which incorporated *W*. *arrhiza*, valuable insights into the evolutionary dynamics affecting populations and species were gained.

## Conclusion

In this study, an analysis of the complete chloroplast genome of *W*. *arrhiza* is provided. The chloroplast genome of *W*. *arrhiza* exhibits resemblances in terms of size, structure, gene composition, GC content, and codon preferences when compared to the typical characteristics observed in land plants and angiosperms. The comparison of Lemnoideae species with *W*. *arrhiza* also demonstrates a significant level of conservation in the chloroplast genome. It also offers details about genes containing nucleotide or intron variations that can be used as molecular markers in the species classification and evolutionary research. The phylogenetic analysis verified that *W*. *arrhiza* is closely related as a sister species to *W*. *globosa*. In summary, the characterization of chloroplast genomic data for *W*. *arrhiza* has provided insights and enriched understanding of the phylogeny of the challenging-to-classify Lemnoideae subfamily using traditional methods.

## Materials and methods

### Plant materials, DNA extraction, and sequencing

The samples of *W*. *arrhiza* were obtained from the Rutgers Duckweed Stock Cooperative (RDSC) located at Rutgers University in New Jersey (http://www.ruduckweed.org/, ruduckweed@gmail.com). The plant collection and use were in accordance with all the relevant guidelines. Among several strains of *W. arrhiza*, voucher number of 7193 was used, which was collected in Masaka, Uganda, located at 0°19′36.3″ S 31°45′13.5″ E. Total genomic DNA was isolated from the whole plant by the modified cetyltrimethylammonium bromide (CTAB) method and quantified by Nanodrop spectrophotometer (Thermo Fisher Scientific, ND-1000) and Qubit Fluorometer (Invitrogen, Thermo Fisher Scientific, Qubit 4). The paired-end libraries were constructed using the MGI Eazy FS DNA Library Prep Kit, with an insert length of 350 bp. Sequencing was performed using reads of 150 bp on the MGI DNBSEQ-G50 second-generation sequencing platform, resulting in a total of approximately 37.82 GB of raw reads being generated.

### Chloroplast genome assembly and annotations

GetOrganelle v1.7.5.3^50^ was utilized to assemble the chloroplast genome using following command (get_organelle_from_reads.py -1 left.fq -2 right.fq -k 21,45,65,85,105 -t 3 -o result_folder -F embplant_pt), and annotation was done with GeSeq^51^. The circular maps for newly sequenced plastomes were generated using the OGDRAW v1.3.1^52^.

### Repeat sequences analysis

The web application MISA^53^ was utilized to detect microsatellites, known as simple sequence repeats (SSRs). The minimum number of repetitions were set as follows: 10 repeat units for mononucleotide SSRs, 6 repeat units for dinucleotide SSRs, and 5 repeat units for tri-, tetra-, penta-, and hexanucleotide SSRs. Furthermore, the web application REPuter^54^ was used to identify long repeats. This process encompassed detecting forward, reverse, complement, and palindromic repeats with a minimum repeat size set at 30 bp and a Hamming distance of three.

### Codon usage

The chloroplast genome's Protein-Coding Genes (PCGs) and their protein coding sequences (CDS) were extracted from the genbank file using Python's SeqIO object. Following that, the Relative Synonymous Codon Usage (RSCU) was calculated using codonW v1.4.4^55^. Synonymous codons refer to codons encoding the same amino acid, and RSCU assesses the relative frequency of such synonymous codons. An RSCU value greater than 1.00 signifies a relatively higher frequency of codon usage, while values less than 1.00 indicate the opposite.

### Chloroplast genome comparison

The tool mVISTA^56^, with the Shuffle-LAGAN mode, was used to identify variations of whole chloroplast genome sequences using *Wolffia australiana* (MN912638.1, strain 8730) as the reference. It visually represents the similarities and differences among seven species, namely *Wolffia arrhiza* (OQ079152.1), *Wolffia globosa* (MN881100.1), *Wolffia australiana* (NC_015899.1, strain 7733), *Wolffia brasiliensis* (MN850406.1), *Wolffiella lingulata* (NC_015894.1), *Lemna minor* (DQ400350.1), and *Spirodela polyrhiza* (NC_015891.1). This entailed utilizing reference information as annotation notes to gain a deeper comprehension of the observed patterns. Additionally, the genome sequences of seven chloroplasts were aligned using the multiple sequence alignment program, MAFFT v7.520^57^. In this alignment, nucleotide diversity (π, Pi) was calculated using DnaSP (DNA Sequences Polymorphism) v6.12.03^58^, employing a window length of 600 bp and a step size of 200 bp. It was also utilized for the calling of SNPs and InDels. The visualization and comparison of the genes located at the junctions of chloroplast genomes in the seven species were carried out using the web application IRscope^59^.

### Phylogenetic analysis

The phylogenetic analysis encompassed a total of 14 species from the Araceae family, which included *W*. *arrhiza*. To facilitate the comparative analysis, *Zea mays* was chosen as the outgroup. The chloroplast genome information was acquired from GenBank, and Python's SeqIO object was employed for parsing and extracting the protein-coding genes and their corresponding protein sequences, which were shared among 15 species. Afterward, the alignment process was conducted using PRANK^60^, and to select the most suitable amino acid substitution model for the alignment data, ProtTest^61^ was employed. The Beast v1.10.4 software^62^ performed Bayesian-based evolutionary analysis, with the CpREV + G + I substitution model, Yule model for prior tree, and the uncorrelated relaxed clock, widely regarded as the most suitable model for datasets at the species level^63^. The Markov Chain Monte Carlo (MCMC) was also set for 50,000,000 generations. Following that, a maximum credibility tree was constructed using TreeAnnotator v1.10.4, discarding the initial 10% of trees as burn-in using Tracer v1.7.1. The phylogenetic tree was then created using FigTree v1.4.4.

### Supplementary Information

Supplementary Figure S1.

Supplementary Tables.

## Data Availability

The genome sequence data that support the findings of this study are openly available in GenBank of NCBI at [https://www.ncbi.nlm.nih.gov/] under the Accession No. OQ079152.
