# Supplementary figures and images for: Characterization of the complete chloroplast genome of Wolffia arrhiza and comparative genomic analysis with relative Wolffia species
Source: Sci Rep. 2024 Mar 11;14:5873. doi: 10.1038/s41598-024-56394-7 (PMC10928178; doi:10.1038/s41598-024-56394-7)

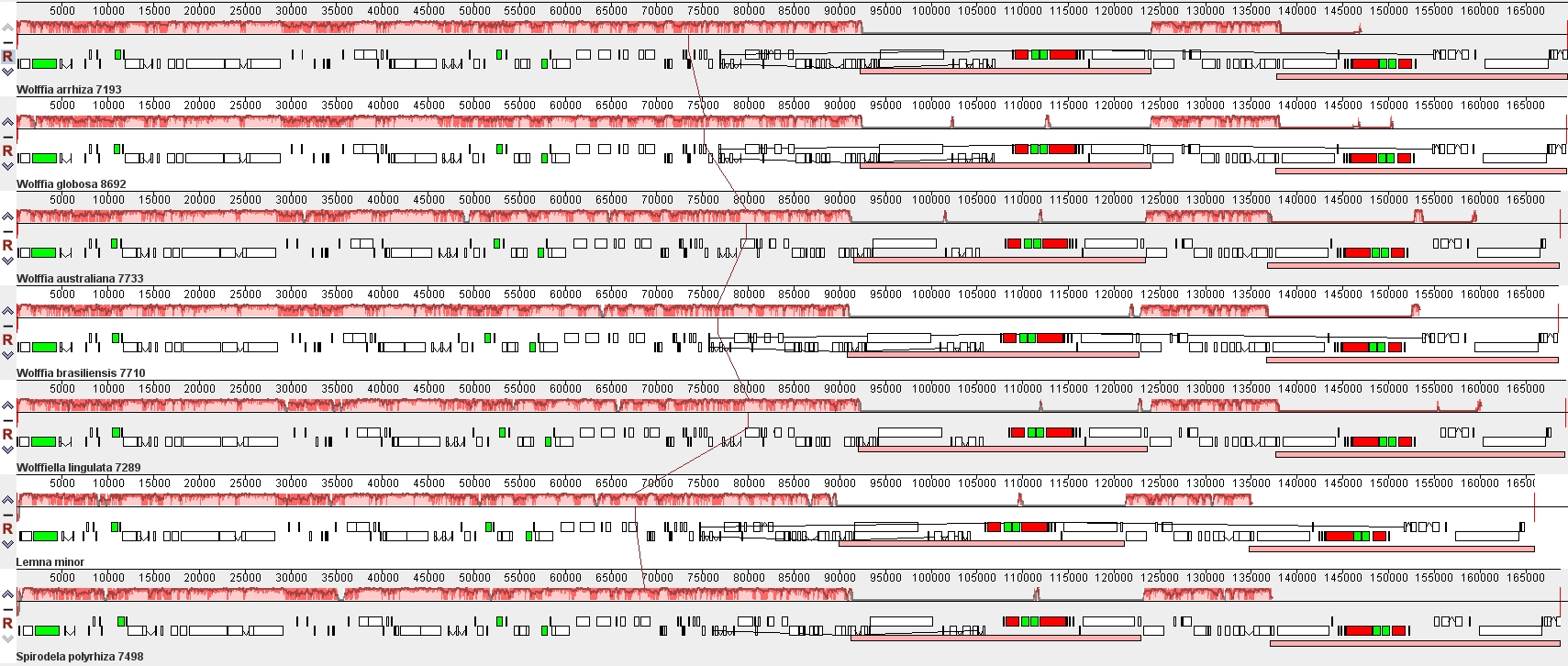

Supplement: Supplementary file 1 — Supplementary Figure S1. [file 41598_2024_56394_MOESM1_ESM.jpg]
